# High-Surface-Area, Emulsion-Templated Carbon Foams by Activation of polyHIPEs Derived from Pickering Emulsions

**DOI:** 10.3390/ma9090776

**Published:** 2016-09-14

**Authors:** Robert T. Woodward, François De Luca, Aled D. Roberts, Alexander Bismarck

**Affiliations:** 1 Department of Chemical Engineering, Imperial College London, South Kensington Campus, London SW7 2AZ, UK; f.de-luca13@imperial.ac.uk; 2 Department of Chemistry, University of Liverpool, Crown Street, Liverpool L69 7ZD, UK; aleddeakin@gmail.com; 3 Polymer and Composite Engineering (PaCE) Group, Institute of Materials Chemistry & Research, Faculty of Chemistry, University of Vienna, Währingerstraße 42, Vienna 1090, Austria; alexander.bismarck@univie.ac.at

**Keywords:** carbon, polyHIPE, carboHIPE, Pickering emulsion, carbonization, microporous, emulsion template

## Abstract

Carbon foams displaying hierarchical porosity and excellent surface areas of >1400 m^2^/g can be produced by the activation of macroporous poly(divinylbenzene). Poly(divinylbenzene) was synthesized from the polymerization of the continuous, but minority, phase of a simple high internal phase Pickering emulsion. By the addition of KOH, chemical activation of the materials is induced during carbonization, producing Pickering-emulsion-templated carbon foams, or carboHIPEs, with tailorable macropore diameters and surface areas almost triple that of those previously reported. The retention of the customizable, macroporous open-cell structure of the poly(divinylbenzene) precursor and the production of a large degree of microporosity during activation leads to tailorable carboHIPEs with excellent surface areas.

## 1. Introduction

Carbonaceous materials possessing hierarchical porosity are desirable owing to a number of advantageous properties, one such example being the coupling of effective mass transfer through macropores and large surface areas derived from both meso- and micropores. High surface areas allow for a large degree of solid-liquid interactions, while effective mass transfer permits rapid access to this surface area throughout the material. More generally, carbonaceous materials are studied prolifically due to their relative chemical inertness and high thermal stability, which has resulted in their consideration as adsorbents [1] or catalyst supports [2], in gas sorption [3], and as electrodes in both Li-ion batteries [4] and double-layer capacitors [5].

The requirement for well-defined porosity within carbon structures has led to the utilization of a range of different precursor materials including carbides [6,7], synthetic organic polymers [8,9], and templated materials [10,11]. One attractive route to hierarchically porous carbon foams is via the carbonization of macroporous polymers templated from high internal phase emulsions (HIPEs). A HIPE is defined as an emulsion containing an internal phase that comprises >74.05% of the total volume of the system, the maximum volume that uniform spheres can close pack without deformation [12]. Polymers are produced from these templates by the polymerization of the continuous, but minority, phase of a HIPE to produce a polyHIPE. Aside from the influence of phase volume ratio and the emulsification method, a great degree of control over the structural properties of a polyHIPE’s macropores is possible simply by varying the choice of emulsion stabilizer in the initial HIPE template. HIPEs are commonly stabilized using standard molecular surfactants, which upon polymerization produce open-cell polyHIPEs, meaning that the polymers contain pore throats which connect the emulsion-templated macropores, producing a more permeable structure. On the other hand, particle-stabilized emulsions, also known as Pickering emulsions, are stabilized by larger particles irreversibly adsorbing at the interface of emulsion [13,14]. Upon polymerization of Pickering HIPEs, closed-cell polyHIPEs containing no pore throats and macropores with larger diameters than those of the surfactant systems are produced [15,16]. However, it was demonstrated by Wong et al. that open-cell polyHIPEs could be produced from Pickering HIPEs via the addition of a small amount of a standard molecular surfactant prior to polymerization, introducing pore throats into Pickering-HIPE derived polyHIPEs [17].

When a charable polyHIPE is produced, it can be used as a precursor to porous carbon foams, or carboHIPEs, which retain the emulsion-templated macroporosity of the polyHIPE while developing micro- and mesopores upon carbonization, creating hierarchical porosity. The production of carboHIPEs has been described in the literature from a wide variety of starting materials, some of which include sulfonated poly(styrene-*co*-divinylbenzene) [18,19], Kraft black liquor [20], lignin [21], tannins [22], and polyacrylonitrile [23]. Recently, we described the production of carboHIPEs from poly(divinylbenzene)HIPEs synthesized from simple Pickering water-in-divinylbenzene (DVB) HIPE templates [24]. Carbonization of these templates gave carboHIPEs in good yields, retaining the emulsion-templated porosity of the poly(DVB)HIPE precursor and producing surface areas of up to 500 m^2^/g. Pickering HIPEs have also been used to create carboHIPEs by producing sacrificial molds in the form of mesoporous macrocellular silica foams [25]. These Si(HIPE)s were infiltrated with a carbon precursor, carbonized, and washed in HF to produce carboHIPEs with surface areas of up to 900 m^2^/g.

In order to try to improve the surface area of carbonaceous materials in general, varying methods of activation have been employed. One method involves the addition of activating agents to charable materials to try to increase the surface area of the resulting carbon by chemical activation during heating. Another involves the carbonization of materials in atmospheres containing small amounts of CO_2_ or H_2_O in order to induce physical activation of the materials. The literature is rich with studies on activation processes, with one of the most popular involving the addition of the chemical activating agent, KOH, to a material prior to carbonization [26]. Previous reports indicate that increases in porosity and surface area brought about by KOH-activation is due to a number of factors including chemical activation by etching of the carbon framework, physical activation by the gasification of the carbon, and carbon lattice expansion by the production of metallic K throughout the carbon framework [26,27,28,29]. It has been demonstrated that the activation of templated mesoporous carbons with well-controlled pore-size distributions led to huge increases in both surface area and total pore volumes by the production of micropores [30,31].

Herein, we report the synthesis of high-surface-area, hierarchically porous carboHIPEs by KOH-activation of poly(DVB) derived from Pickering HIPEs. The successful retention of emulsion-templated macroporosity during activation allows for carboHIPEs with almost triple the surface area of their non-activated (simply carbonized) equivalents. A combination of both silica particles and a small amount of standard molecular surfactant were used in order to create open-cell, charable structures. This increase in surface area, coupled with customizable macropore diameters, opens up opportunities to produce tailorable carboHIPEs with excellent surface areas.

## 2. Experimental

### 2.1. Materials

Calcium chloride (CaCl_2_), divinylbenzene (DVB, 80% containing inhibitor), azobisisobutyronitrile (AIBN), and potassium hydroxide (KOH) were all purchased from Sigma-Aldrich (Dorset, UK) and used as received. Hydrophobic silica particles (HDK grade H20) were kindly provided by Wacker Chemie (Bracknell, UK), and Hypermer 2296 was kindly provided by Croda (Leek, UK). Both products were used as received. Silver paint used for SEM imaging was purchased from Agar Scientific (Stansted, UK).

### 2.2. Preparation of HIPEs and Subsequent poly(DVB)HIPEs

The synthesis of poly(DVB)HIPEs from Pickering water-in-DVB HIPEs has been described previously [24]. For a typical formulation, hydrophobic silica particles (120 mg, 3 wt %) were added to DVB (4 mL), and the sample was shaken by hand for ~5 min until the particles were well dispersed in the monomer. AIBN (1 mol % with respect to the monomer, 47 mg) was then added and the mixture stirred using a vortex mixture ((VortexGenie 2, Scientific Industries, Bohemia, NY, USA), speed setting ‘3’, equating to roughly 600 rpm) before an aqueous CaCl_2_ solution (10 g/L, 16 mL) was added slowly over 20 min while still stirring using the vortex mixer at the same speed setting. After the addition of the aqueous phase was complete, the HIPE was stirred more vigorously (speed setting ‘10’, roughly 3000 rpm) for a further 5 min before the surfactant Hypermer 2296 (0.2 mL, 5 vol %) (Croda, UK) was added with respect to the initial monomeric phase, after which the HIPE was gently agitated by hand for 10 s. To create poly(DVB)HIPEs with smaller macropores, the HIPE was stirred at either 1000 rpm or 3000 rpm for 10 s at this point in the process, in place of gentle agitation by hand. HIPEs were then transferred into a 15 mL free standing polypropylene centrifuge (Falcon) tube (VWR, Radnor, PA, USA) and heated at 70 °C for 24 h in a convection oven to initiate polymerization. After polymerization, the Falcon tube containing the polyHIPE was cut into roughly 1 cm^3^ cylinders using a band saw before the polyHIPE was removed from the tubes and washed three times in an ethanol bath for a total of at least 6 h. The cylinders were then dried in a vacuum oven at 110 °C overnight.

### 2.3. Preparation of carboHIPEs and Activated carboHIPEs

Samples of poly(DVB)HIPE were prepared for either carbonization or carbonization in the presence of a chemical activator. All samples were weighed before heating to 800 °C under a N_2_ atmosphere at a ramp rate of 2 °C/min. Once 800 °C was reached, samples were held at this temperature for 1 h before the furnace was allowed to cool to room temperature overnight (remaining under a N_2_ atmosphere). Samples being prepared for chemical activation were also weighed before being soaked in an aqueous KOH solution (either a 10 wt % or a 30 wt % solution), after which they were carefully removed and placed in a convection oven at 70 °C to dry overnight. After drying, samples were weighed again to determine how much KOH was deposited before being placed in the furnace and carbonized using the same process as the non-activated carboHIPEs. When investigating tailored average macropore diameters in activated carboHIPEs, all poly(DVB)HIPEs were immersed in a 30 wt % KOH solution before subsequent drying and carbonization as described above.

### 2.4. Characterization

Gas sorption analyses were performed on a Micromeritics 3Flex Surface Characterization Analyzer (Micromeritics, Atlanta, GA, USA) at −196 °C. Samples were degassed in situ under vacuum (around 0.0030 mbar) at 150 °C for at least 4 h, prior to measurement. SEM images were either taken on a variable pressure SEM (JEOL JSM 5610 LV (0.5–35 kV), Tokyo, Japan) or, in the case of the high-resolution SEM, images were taken using a high-resolution field emission gun SEM (FEGSEM (5 kV, InLens detector)) (Leo Gemini 1525 coupled with a SmartSEM software interface, Carl Zeiss NTS Ltd., Cambridge, UK). All polyHIPE samples were fixed on Al stubs (Agar Scientific Ltd., Stansted, UK) using carbon tape to attach samples securely. The stubs were then sputtered with chromium (10 nm) and, taking care not to contaminate the sample, a small amount of silver DAG paint was used to provide a conductive bridge between the carbon tape and the Al stub. Image analysis, such as measuring the average macropore diameter of polyHIPEs, was carried out using the image software ImageJ (version 1.48, National Institutes of Health, Bethesda, MD, USA) [32]. The percentage porosity (P) was determined from both the envelope (*ρ_e_*) and skeletal density (*ρ_s_*) via the equation P = (1 − *ρ_e_*/*ρ_s_*) × 100%.

### 2.5. Research and Discussion

**Figure 1:**
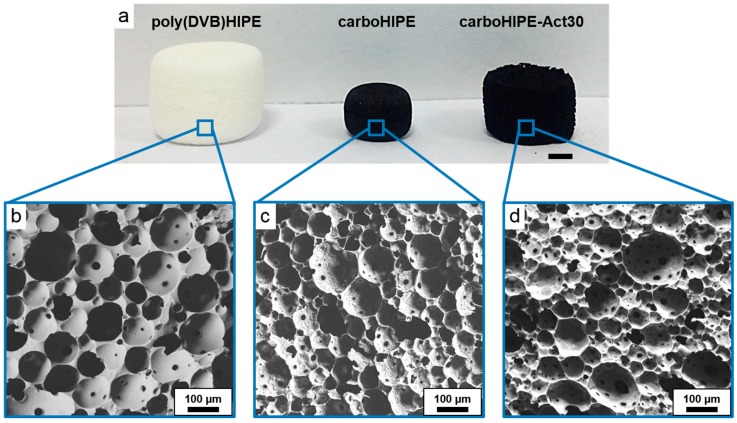
(**a**) Photograph of a poly(DVB)HIPE, carboHIPE, and a carboHIPE-Act30; (**b**–**d**) SEM images of a poly(DVB)HIPE, carboHIPE, and a carboHIPE-Act30, respectively. The scale bar in the photograph represents 2 mm.

Water-in-DVB emulsions were prepared using a combination of silica particles and standard molecular surfactants as stabilizers. The use of the Pickering emulsifiers was crucial as we previously demonstrated that poly(DVB)HIPEs derived from solely surfactant-stabilized HIPEs did not survive carbonization [24]. After curing the emulsions for 24 h at 70 °C, all white poly(DVB)HIPEs were produced (Figure 1a), which displayed emulsion-templated macroporosity with an average diameter of 82 μm (Table 1), in a similar range to other polyHIPEs produced from Pickering HIPEs [16,33]. As a mixed surfactant system was used, pore throats were created in the resulting poly(DVB)HIPE, producing the desired open-cell structure (Figure 1b). Poly(DVB)HIPEs were carbonized at 800 °C in an inert N_2_ atmosphere and yielded carbon foams, or carboHIPEs (Figure 1a). The carboHIPEs retained the cylindrical shape of poly(DVB)HIPE precursors well, albeit with a significant volume loss of between 73% and 76%. The emulsion-templated macropores also survived carbonization to yield an open-cell carboHIPE, showing a decrease in their average diameter of 20 μm to 62 μm. (Figure 1c).

**Figure 2:**
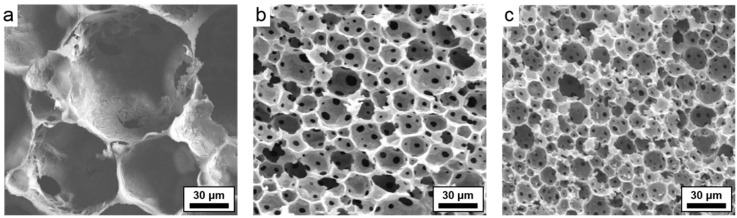
SEM images of various carboHIPE-Act30s with different average macropore diameters, produced by stirring the HIPEs at different speeds prior to polymerization. (**a**) Gently agitated by hand; (**b**) 1000 rpm for 10 s on a vortex mixer; and (**c**) 3000 rpm for 10 s on a vortex mixer.

Varying levels of activation of poly(DVB)HIPEs were achieved by submerging poly(DVB)HIPEs in KOH solution of varying concentrations for two hours under gentle agitation, prior to carbonization. The poly(DVB)HIPEs were then dried in an oven and carbonized in a similar procedure to the non-activated carboHIPEs. Activated materials will be denoted by the concentration of the KOH solution in which they were submerged; for example, in carboHIPE-Act10, the ‘Act’ refers to activation and ‘10’ indicates that the poly(DVB)HIPE precursor was exposed to a 10 wt % KOH solution. Activation of poly(DVB)HIPEs appeared to lead to slightly larger carboHIPEs than simple carbonization, with volume losses in activated materials ranging between 50% and 61% (Figure 1a), although this was admittedly hard to quantify in many samples due to the more irregular shapes and slightly more brittle nature of activated materials. The higher retention in volume may be due to the increased evolution of gas exerting outward pressure on structures during activation, or intercalated K in the carbon framework preventing further collapse. The emulsion-templated macropores were successfully retained during activation (Figure 1d) and showed a slightly reduced average macropore diameter of 74 μm for carboHIPE-Act30, in good agreement with the reduced volume loss in comparison to simple carbonization in the absence of KOH. In order to demonstrate the tailoring of macropore size in activated carboHIPEs, standard water-in-DVB HIPEs were stirred at varied rates after the addition of the Hypermer 2296 surfactant, prior to curing and activation. The samples were either gently agitated by hand (as is the case in the standard procedure), or stirred for 10 s at 1000 rpm or 3000 rpm using a vortex mixer. Average macropore diameters of 74 ± 30 μm, 18 ± 5 μm, and 11 ± 4 μm were obtained for the standard carboHIPE-Act30, and those stirred at 1000 and 3000 rpm, respectively (Figure 2), demonstrating controllable macropore size in activated carboHIPEs.

**Figure 3:**
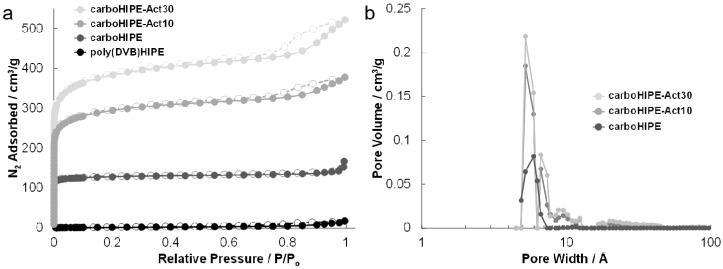
(**a**) N_2_ adsorption/desorption isotherms of all materials including a poly(DVB)HIPE, a carboHIPE, and carboHIPEs with varying degrees of activation (carboHIPE-Act10 and carboHIPE-Act30). Filled shapes show adsorption while empty shapes show desorption; (**b**) Non-local density functional theory pore size distribution for a carboHIPE, carboHIPE-Act10, and carboHIPE-Act30.

Gas sorption analysis was performed on samples, applying the BET model for surface area measurements and determining the micropore volume using the t-plot method. The native poly(DVB)HIPE had a low surface area of 8 m^2^/g due to the presence of some mesoporosity within the structure [24,34]. All surface areas showed huge increases upon carbonization or activation, with nitrogen adsorption displaying a type I isotherm in all cases (Figure 3a), with a steep N_2_ uptake at low pressures, indicative of a predominantly microporous structure, as outlined by IUPAC classification [35]. The surface areas of carboHIPEs increased significantly with the addition of KOH prior to carbonization (Table 1), producing excellent surface areas of up to 1456 m^2^/g for carboHIPE-Act30. Both the total pore volume and the micropore volume also improved dramatically upon carbonization or activation, increasing from no notable microporosity in the poly(DVB)HIPE to an excellent micropore volume of 0.544 cm^3^/g and a total pore volume of 0.791 cm^3^/g in carboHIPE-Act30. Pore size distributions show this increase in micropore volume with a large peak at ~5 Å after both carbonization and activation, with activated samples appearing significantly more microporous (Figure 3b). By increasing the concentration of the initial KOH solution used to deposit the activating agent, both the surface area and the micropore volume could significantly increase (Figure 3, Table 1) without any significant structural damage occurring to the emulsion-templated macropores (Figure 1, Table 1).

**Figure 4:**
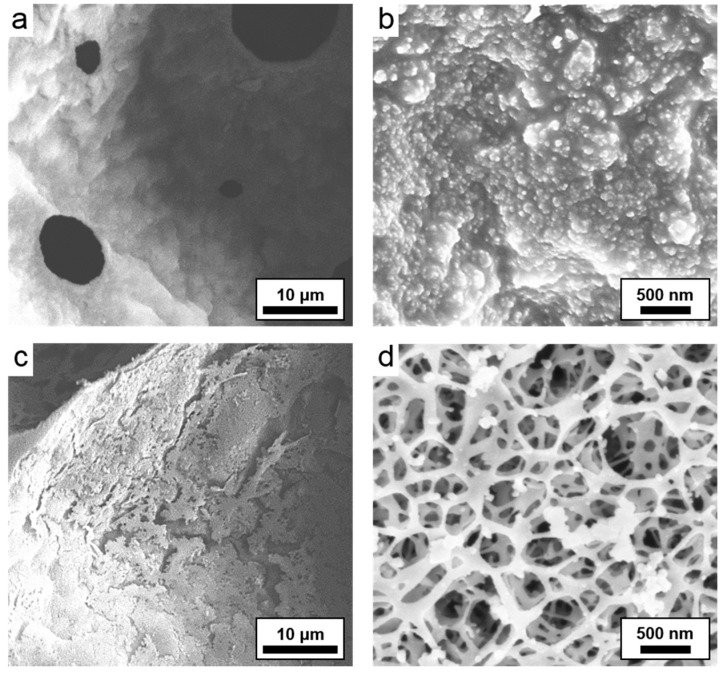
SEM images. (**a**,**b**) show a typical non-activated carboHIPE; and (**c**,**d**) show a typical activated carboHIPE-Act30.

In order to further investigate the effect of the activating agent on the emulsion-templated structure and what may lead to the large increases in surface area, high magnification SEM was performed. When no activation agent was used during carbonization, the surface of the carboHIPE looked relatively smooth and unbroken with the exception of the pore throats in the images (Figure 4a,b). However, in carboHIPE-Act30, the surface was cracked and appeared more damaged after activation in comparison to carbonization with no KOH (Figure 4c,d). Considering the high magnification images of both the carboHIPE and carboHIPE-Act30, a much more porous structure is observed for the latter (Figure 4), with the visible pores mainly in the macropore region (>50 nm in diameter). This newly formed porous structure may allow better access to the increased amount of micropores throughout the structure, resulting in much higher surface areas.

**Figure 5:**
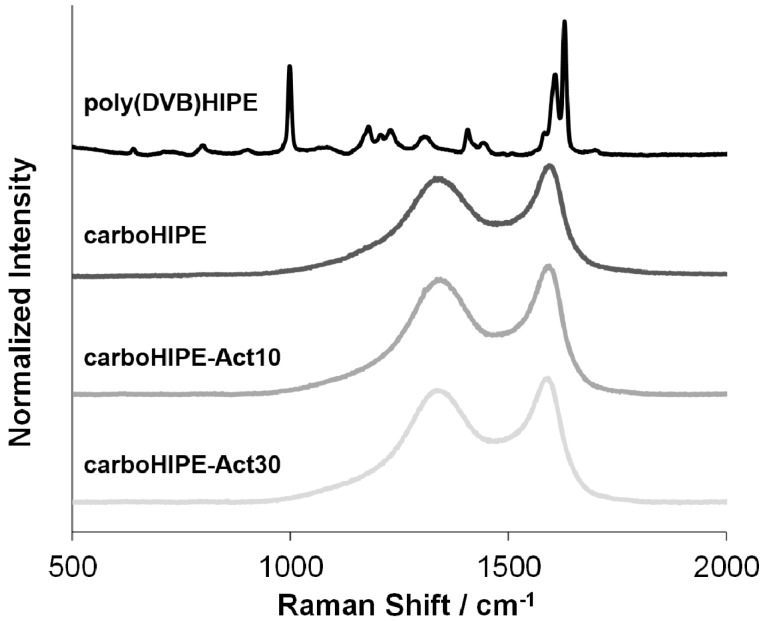
Raman spectroscopy of a poly(DVB)HIPE, a carboHIPE, and activated carboHIPEs.

Lastly, the degree of graphitization between samples was probed using Raman spectroscopy (Figure 5). It is clear from the spectra that carbonization/activation led to the formation of D and G modes (1350 cm^−1^ and 1582 cm^−1^, respectively) and the loss of the typical fine structure of the poly(DVB)HIPE in the Raman spectrum [36]. The formation of these broad D and G peaks is indicative of disordered graphitic carbonaceous structures [37], typical for carbonization at relatively low temperatures [38]. The D to G peak intensity ratio is around 0.9 for all samples, suggesting that the degree of graphitization is independent of the degree of activation in the carboHIPEs.

## 3. Conclusions

By simple immersion and subsequent drying of Pickering-emulsion derived poly(DVB)HIPEs in inexpensive KOH solutions, carboHIPEs containing hierarchical porosity and surface areas of up to 1456 m^2^/g were easily produced upon carbonization. This addition of this simple step almost triples both the resulting surface area and micropore volume of carboHIPEs in comparison to poly(DVB)HIPEs carbonized in the presence of no activating agent. The degree of microporosity was controlled by varying the amount of KOH present during carbonization. The activation process was not detrimental to the emulsion-templated porosity of the poly(DVB)HIPEs, and it was demonstrated that the size of the macropores could also be dictated in activated carboHIPEs by controlling the amount of energy input after the addition of the standard surfactant. The retention of the macropores along with the production of large micropore volumes opens up potential for efficient mass transfer of electrolytes to the increased surface areas of these carbon foams, meaning they may have potential as novel monolithic electrodes in supercapacitor devices and will be investigated further. The tunable porosity of these materials on both the macro- and the micropore scale could also lead to their use as efficient adsorbents for the removal of organic pollutants, or as high surface area catalyst supports.

## Figures and Tables

**Table 1:** Macropore diameter, BET surface area, micropore volume, total pore volume, porosity, and char yield of a poly(DVB)HIPE, a carboHIPE, and activated carboHIPEs.

Sample	Average Macropore Diameter (μm) ^a^	Surface Area (m^2^/g) ^b^	Micropore vol. (cm^3^/g) ^b^	Total Pore vol. (g/cm^3^) ^b^	Porosity (%) ^c^	Char Yield (%) ^d^
poly(DVB)HIPE	82 ± 42	8	0	0.021	86	N/A
carboHIPE	62 ± 28	521	0.268	0.223	95	22
carboHIPE-Act10	72 ± 26	1123	0.432	0.572	97	13
carboHIPE-Act30	74 ± 30	1456	0.554	0.791	97	12

^a^ Measured using image analysis software; ^b^ Calculated from N_2_ sorption isotherms at 77 K; ^c^ Calculated using both the skeletal and envelope density of monoliths and ^d^ Mass yield after carbonization, relative to original poly(DVB)HIPE.
